# A realist synthesis of the effect of social accountability interventions on health service providers’ and policymakers’ responsiveness

**DOI:** 10.1186/2046-4053-2-98

**Published:** 2013-11-07

**Authors:** Elsbet Lodenstein, Marjolein Dieleman, Barend Gerretsen, Jacqueline EW Broerse

**Affiliations:** 1 Athena Institute for Research on Innovation and Communication in Health and Life Sciences (VU University) and Royal Tropical Institute (KIT), De Boelelaan 1085, Amsterdam, HV 1081, The Netherlands; 2 KIT Development, Policy and Practice, Royal Tropical Institute (KIT), PO Box 95001, Amsterdam, HA 1090, The Netherlands; 3 Athena Institute for Research on Innovation and Communication in Health and Life Sciences (VU University), De Boelelaan 1085, Amsterdam, HV 1081, The Netherlands

**Keywords:** Citizen participation, Social accountability, Responsiveness, Health service delivery, Health policymaking, Realist synthesis

## Abstract

**Background:**

Accountability has center stage in the current post-Millennium Development Goals (MDG) debate. One of the effective strategies for building equitable health systems and providing quality health services is the strengthening of citizen-driven or social accountability processes. The monitoring of actions and decisions of policymakers and providers by citizens is regarded as a right in itself but also as an alternative to weak administrative accountability mechanisms, in particular in settings with poor governance. The effects of social accountability interventions are often based on assumptions and are difficult to evaluate because of their complex nature and context sensitivity. This study aims to review and assess the available evidence for the effect of social accountability interventions on policymakers’ and providers’ responsiveness in countries with medium to low levels of governance capacity and quality. For policymakers and practitioners engaged in health system strengthening, social accountability initiatives and rights-based approaches to health, the findings of this review may help when reflecting on the assumptions and theories of change behind their policies and interventions.

**Methods/Design:**

Little is known about social accountability interventions, their outcomes and the circumstances under which they produce outcomes for particular groups or issues. In this study, social accountability interventions are conceptualized as complex social interventions for which a realist synthesis is considered the most appropriate method of systematic review. The synthesis is based on a preliminary program theory of social accountability that will be tested through an iterative process of primary study searches, data extraction, analysis and synthesis. Published and non-published (grey) quantitative and qualitative studies in English, French and Spanish will be included. Quality and validity will be enhanced by continuous peer review and team reflection among the reviewers.

**Discussion:**

The authors believe the advantages of a realist synthesis for social accountability lie in the possibility of overcoming disciplinary or paradigmatic boundaries often found in public health and development. In addition, they argue that this approach fills the knowledge gap left by conventional synthesis or evaluation exercises of participatory programs. Finally, the authors describe the practical strategies adopted to address methodological challenges and validity.

## Background

The Millennium Development Goals (MDGs), international goals agreed at the United Nations (UN) Millennium Summit in 2000 and covering the period from 2000 to 2015, have contributed to increased investments in basic services, such as health. The international community, led by the UN and in particular the World Health Organization (WHO), is currently involved in a debate about the nature of the post MDG agenda for health [1]. It is suggested that the new development framework for health emphasizes people-centered and rights-based approaches with a focus on building equitable, accountable and sustainable health systems [2]. The respect, promotion and fulfillment of the right to health by governments and its translation into local practice is a key challenge of the new era. In this context, an important question is how to hold states accountable for meeting their commitments to improving, for example, equality and non-discrimination in health care. One way of doing this might be to strengthen the role of citizens in the monitoring and review of the actions and decisions of policymakers and providers at international, regional, national and local levels [3].

Social accountability (also called citizen-driven accountability or bottom-up accountability) refers to the strategies, processes or interventions whereby citizens voice their views on the quality of services or the performance of service providers or policymakers who, in turn, are asked to respond to citizens and account for their actions and decisions. These efforts may be supported by governments, civil society, media or other actors [4,5]. The approach aims to enhance the responsiveness of health providers and policymakers to citizens’ demands. The relevance of social accountability can be analyzed from two perspectives. From an institutional economics perspective, social accountability is seen as complementary to administrative or bureaucratic accountability, which has government-led or regulatory mechanisms of monitoring. From this perspective, one of the key challenges to accountability in health systems is the principal-agent issue, namely that national state actors cannot be fully held responsible for performance because of the multiple, hierarchical levels of delegation. Front-line health workers in primary health care operate at the lowest level in the hierarchy and have a certain degree of discretion in their decisions and actions, which cannot be controlled by the principal [6]. In settings with a poor regulatory capacity, government-led monitoring and accountability mechanisms hardly exist and there is an increasing expectation that social accountability might be able to compensate for that gap and exercise some form of control [7,8]. Parallel to this institutionalist perspective, social accountability has roots in several broader trends in development, including in rights-based approaches and participatory governance [9]. These social movement approaches emphasize the voice, agency and collective action dimensions of accountability [10,11].

Depending on the perspective, the expected results of social accountability initiatives can vary, but include a reduction in corruption; better governance and policy design; enhanced voice, empowerment and citizenship of marginalized groups; responsiveness of service providers and policymakers to citizens’ demands and, ultimately, the achievement of rights, health and developmental outcomes [4,10,12].

Citizen-driven accountability has been promoted over the past few years and some positive results and critical lessons have been reported for service delivery [4,6,13-15]. There remain, however, a number of questions regarding how social accountability interventions are conceptualized, how they work in practice across contexts and how they can be evaluated. The aim of this study is to review and assess the available evidence of the effect of social accountability interventions on providers’ and policymakers’ responsiveness in health service delivery and policymaking in countries with medium to low levels of governance capacity and quality. The reviewers are particularly interested in how social accountability interventions work and under which conditions they lead to specific outcomes.

This realist synthesis constitutes the first phase in a larger research program in sub-Saharan Africa on social accountability in maternal health service delivery. This program is being implemented in countries with poor governance capacity and quality, including those in post-conflict or fragile settings, such as Burundi, the Democratic Republic of Congo, Mali and Guinea. The results of this synthesis will inform the empirical research phase of this program; it will in particular support the identification and selection of case studies and inspire the development of evaluation methods. In addition, the results of the proposed review may be useful for development organizations engaged in health system strengthening, social accountability initiatives and rights-based approaches to health, both internationally and locally. This includes non-governmental organizations (NGOs), civil society organizations (CSOs), and their funders and networks. The findings may support reflection on the design and implementation of development programs by reporting on the challenges social accountability interventions encounter in specific contexts. From a conceptual perspective, this review will support the positioning of social accountability dynamics in the wider accountability debate in basic service delivery.

### A realist perspective

An evaluation of the effectiveness of social accountability interventions faces a number of methodological challenges, of which an important one is related to complexity. For example, interventions, such as citizen complaint hotlines or citizen scorecards that require the public disclosure of performance, are expected to increase the incentives for service providers to perform. However, interventions take place in existing interaction spaces where there may be other formal and informal incentives for citizens to voice their concerns and public agents to listen to those concerns [11,16-18]. For example, health policymakers or service providers may be triggered to become responsive by feelings of moral obligation or by fear of sanctions from superiors or a combination of both [17,19]. The objects of citizen engagement are very diverse as are the participants in citizen engagement strategies. For example, the emergence of a citizen voice may depend on the frequency of service use or the availability of provider choice [20]. In addition, voice and responsiveness dynamics will play out differently for claims of neglect and avoidable death than for demands to reduce waiting times [13]. Health providers and policymakers may respond differently to men and women and to poor and non-poor demands, which Goetz and Gaventa (2001) call ‘exclusive responsiveness’ [21-24].

As these examples illustrate, the outcomes of interventions are highly dependent on human agency and context and interventions do not linearly produce outcomes. Many sources of complexity and subtle behavioral dynamics remain hidden in standard methods of systematic review, which focus on the assessment of outcomes (does it work?). In complex interventions, such as social accountability, standard systematic reviews have limited value for transferring lessons from one context to another [25]. An alternative is to distill how, for example, health system characteristics or intervention components (context) influence individuals to act in certain ways (be responsive to citizens or not) to produce certain outcomes (improved quality). This alternative method of realist review can, to a larger extent than standard systematic reviews, explain how context influences the outcomes of interventions [26,27]. This method seems most appropriate for our evaluation of social accountability interventions.

Realist synthesis is rooted in realism and critical realism within philosophy and the social sciences. It is a logic of inquiry that is theory driven and that facilitates an explanation of what works, for whom, in what circumstances and in what respects [28]. Philosophers such as Bhaskar, Archer, Merton, Campbell and Popper have inspired the development of a realist approach (see Pawson, currently a leading author in the field [29]).

Critical realism as explained by Bashkar begins with the notion that scientific inquiry is more than observation and the measurement of facts. It starts from an assumption about complexity [29]. Social phenomena are built from the actions of actors and by their interpretation of these phenomena. Actors are constrained or enabled by social structures. The interplay between agents and structures influences the working of interventions, and research should try to understand how agent–structure interactions produce social change [25]. The realist approach proposes a systematic integration of contextual analysis in the synthesis and evaluation of interventions. It asserts that it is not the intervention that generates outcomes, but rather that the mechanisms in certain contexts produce outcomes [29]. Therefore, the influence of context and the mechanisms it triggers, implies that an intervention might work well in one context but not in another [26]. Mechanisms refer to the reasoning and behavior of participants and stakeholders in an intervention. Context-mechanism-outcome (C-M-O) configurations produce a ‘generative explanation for causation’ [26]. Interventions, such as those promoting social accountability, can influence the course of change within society, but will never on their own lead to change [25].

A realist synthesis aims to provide explanations for successes or failures but does not aim to generate judgments [25,26,28]. Realist synthesis is grounded on program theory, which is crucial for moving beyond ad hoc or piecemeal explanations [29]. Through a synthesis process, the program theory or theories are tested and further refined by identifying how contextual factors (C) influence the production of outcomes (O) through the triggering of specific mechanisms (M) in the form of context-mechanism-outcome (C-M-O) configurations [28,29]. These C-M-O configurations may constitute patterns, called demi-regularities, which in turn support or contradict program theories. Program theories can be further synthesized and generalized to form middle-range theories (MRTs). MRTs help to develop a level of abstraction needed to understand the diversity of outcomes across contexts [25]. In this review, the processes through which social accountability leads to change might have some universal characteristics, although their application will be adapted to local contexts.

## Methods/Design

### Research framework and research questions

**Figure 1:**
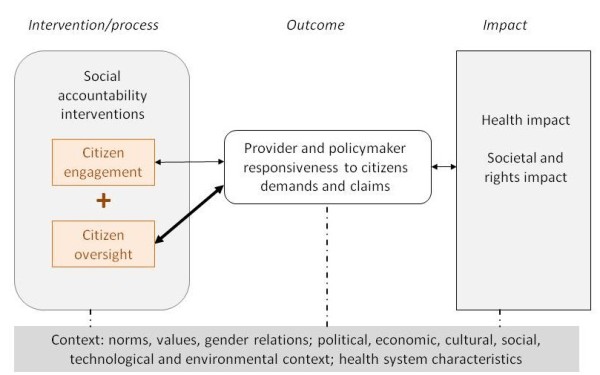
Preliminary program theory.

After an initial literature review, a preliminary program theory has been developed for this review. It is based on the hypothesis that social accountability in health service delivery results in providers’ and policymakers’ responsiveness to citizens’ preferences and claims, and ultimately in rights and developmental outcomes. This is schematically presented in Figure 1. In terms of social accountability interventions, we differentiate between citizen engagement strategies (participation and voice without formal means of enforcement) and citizen oversight strategies (which usually involve monitoring, enforcement and sanctioning). It is argued that, under certain conditions, citizen engagement strategies can engender the responsiveness of service providers and policymakers. However, these are considered to be regular political processes and not necessarily social accountability interventions [10,16]. Citizen engagement strategies are more likely to improve responsiveness if they are backed by formal accountability mechanisms and oversight by civil society, the media, judiciary or governmental actors [5,10,16,17,30,31]. Responsiveness is defined as the extent to which a health provider or health policymaker demonstrates receptivity to the ideas and concerns raised by citizens by implementing changes to the decision-making or management structure, culture, policies or practices [14,24]. Increased responsiveness is ultimately expected to result in a stronger health and rights impact, such as an increase in user satisfaction or service utilization, or a decrease in the prevalence of disease, in particular for previously excluded groups [12]. Social accountability interventions, responsive behavior, and the health and rights impact are influenced by contextual factors, such as societal values, gender relations, levels of political stability and health system characteristics (see Table 1 for more detailed examples of levels and definitions of outcomes and contextual factors). In this review, the focus will be on the levels of the process and outcomes for responsiveness. Other outcomes (for example for citizen voice or patient information) and the impact on health and rights will be recognized but the key focus of the analysis and synthesis will be on the interaction between the intervention and its outcomes at the providers’ and policymakers’ level.

**Table 1:** Framework for data extraction

**Concept**	**Definition**	**Examples**
***Realist review concepts***		
Context	Actors or factors that are external to the intervention, occurring independently of the outcome or influencing the outcome [32]. “Context can also be understood as anything that can trigger and/or modify the behavior of a mechanism” [33].	*Issue/problem:* The issues citizens are confronted with or about which they express demands, claims, suggestions, opinions, preferences and so on. For example, they may be about the quality, acceptability, accessibility or availability of health services and policies, equality (gender, class or ethnicity) or particular medical aspects of the citizen–provider interaction (disease recognition, treatment and so on) [34]. The issues at stake may also be about equity, discrimination, exclusion and demands for changes in the underlying power relations.
		*Political/institutional factors:* The nature and strength of civil society; models of deliberation, information, capacities and awareness of citizens; the strength of the health system; the capacities, power and incentives of providers and policymakers; the nature and scope of existing social accountability relations; the legal context of citizen engagement; media and press; the historical context; pre-intervention activism; political economy factors; formal and informal political processes; general views on participation; cultural norms and the history of the community where the intervention is implemented [33,34].
		*Other interventions:* Other participatory interventions, government-led accountability initiatives, initiatives to strengthen citizen participation in general or for health.
Mechanism	Interplay between structure and agency, how the social structure interacts with individual or group agency. Mechanisms can be found at individual, group, organizational or societal levels. They are psychological or social explanations of behavior. They may be the cognitive or emotional responses of people who want to participate (or not) in an intervention. They are not intervention strategies, which are intentional measures taken by program implementers [32,33]. Mechanisms are defined as elements of the reasoning of the actor facing an intervention [26,29].	In documents they may be referred to as barriers or facilitators and/or successful or unsuccessful elements of the intervention and its outcomes. They are most likely presented in the discussion or lessons learned section. Mechanisms may be expressed through interpretations, considerations, decisions or behaviors of humans, including the authors of a document. Within the factors being discussed, the behavioral elements will need to be distilled. In the context of citizen engagement and social accountability, mechanisms may refer to triggers that make a citizen or a provider decide or act in favor or not in favor of the intervention. His/her considerations may be a simple cost–benefit analysis, the expected success for the individual or the collective, the mandate of the activity, the trust in, and behavior of the facilitators, and so on.
Outcome	Intended or unexpected outcome of an intervention. It can be defined as intermediate or final [33]. It can be reported at the individual, organizational, institutional, policy or legislative level. It can be both positive and negative.	The reviewers distinguish between process outcomes and health, rights and development outcomes (called impact). Process outcomes can be found on the citizens’ side and on the providers’ side. For citizens, outcomes can be levels of empowerment, voice, agency, awareness, knowledge, satisfaction, trust. Change in providers’ and policymakers’ responsiveness can be levels of accountability: receptivity, responsibility, recognition of issues and concerns, inclusion, acceptance, discrimination, coherence, cohesion, confrontation, conflict, trust, quality and performance (e.g. related to accessibility, acceptability) reflected in changed behavior, policies and practices. Impact refers to health outcomes such as increased utilization of or attendance at health services, prevalence and treatment rates.
C-M-O configurations	A C-M-O can pertain to a whole program or parts of it and one C-M-O can be embedded within another. They can also be configured in a series where the outcome of one C-M-O constitutes the context for the next [33].	Example with responsiveness as an outcome: a set of mechanisms (M) that influence health providers’ and policymakers’ responsiveness vis-à-vis citizens preferences and demands (O) that are triggered by criticism in mass media and contextual factors such as the openness of the health system to public opinion (C).
Social accountability	Social accountability is sometimes understood in terms of tools, in other instances, it refers to social and political processes of the citizen–state interaction. The reviewers will consider the effectiveness of the process; it will need to be analyzed in the wider intervention context and the context of the processes of citizen engagement and oversight mechanisms that are part of it. Citizen oversight is the ability of citizens to influence the quality or equity of health services and policies through the use of, or supported by, pressure or accountability mechanisms. There is a notion of collective action. The more activist definition is ‘collective challenges to medical policy and politics, belief systems, research and practice that include an array of formal and informal organizations’. Challenges are to political power, professional authority and personal and collective identity’ [35].	See interventions below.
Social accountability intervention	For this review, the authors distinguish between two types of interventions (see inclusion criteria):	
	1. Explicit social accountability interventions are interventions that aim to empower citizens to articulate, voice and express their concerns regarding service delivery with the aim of transforming provider organizations or policymaking institutions. They most probably are initiated or driven by citizens and they most likely involve collective actions by associations or groups of citizens (and not by individuals).	Examples of explicit social accountability interventions: Strategies aimed at enhancing citizen engagement and oversight (see above) through approaches such as information campaigns about rights and entitlements, performance standards, collecting and reviewing evidence, collective monitoring (public hearings, opinion polls, citizens’ juries, community scorecards, social audits, citizen report cards and so on), mobilization and advocacy [4,10]. They may also include more informal or spontaneous actions such as protest, petitioning, strikes, strategic non-participation or the use of cultural symbols [34]. These processes may be facilitated by an intermediary structure (Community Health Worker, non-governmental organization or civil society organization, health committee or local council). Decentralization and devolution reforms will be included as they aim explicitly at strengthening local citizen–state relations, voice and responsiveness.
	2. Implicit social accountability interventions are strategies that citizens, health providers or policymakers undertake to influence or change their relation with citizens. Rather than having an explicit aim to strengthen citizen voice and accountability, these elements of social accountability are reported or observed in the process or the outcomes. They are most likely initiated by governmental agents or providers themselves and most likely concern individual patients.	Examples that implicitly address (elements of) social accountability: 1) providers and policymakers may introduce participatory planning, monitoring and evaluation techniques, share information or seek citizens’ opinions (client satisfaction surveys, participatory maternal death audits or complaint mechanisms); 2) outreach and health education strategies that include interventions that aim to raise awareness and change health behavior. They are most likely focused at increasing access and coverage 3) health insurance and community financing using community resources (land, labor or money) to increase access or reduce the costs of providing services. In these cases, the main focus of the intervention is on financing, not on citizen–provider–policymaker relations [15]. These three examples of interventions will be excluded in the first round of this review because the reviewers expect that they do not aim at, or result in, citizen engagement or oversight, or provider and policymaker responsiveness.
Actors	Studies may define the actors involved differently, but they can probably be identified as being on the citizen side or on the provider or policymaker side.	A distinction will be made between the initiators/implementers and target groups/participants as well as the intermediary structures if relevant.
Program theory	A mostly implicit set of assumptions that steers the choice and design of an intervention. It is the black box between the intervention and the outcome; it explains how and why the intervention is expected to produce outcomes [25,32].	The reviewers will use their own preliminary program theory to assess the evidence in the literature. As part of the assessment however, they will also report on the program theory of the interventions studied (see review questions).
Middle-range theory (MRT)	‘A program theory is considered middle range when it is capable of retaining its relevance across multiple cases and in differing contexts’ [33].	

The central review questions are: when and how do social accountability interventions influence providers’ and policymakers’ responsiveness in health service delivery in countries with medium to low levels of governance capacity and quality?

More specifically:

● What is the expected chain of results of the social accountability intervention (program theory)?

● What are the reported (favorable and unfavorable) outcomes of the interventions? At what levels (individual, organizational, societal and so on)? In particular, what are the reported responsiveness outcomes?

● Who is reported to be (both positively and negatively) affected by the intervention and its outcomes?

● What mechanisms and contextual elements help to explain the outcomes of the intervention?

By answering these questions through a realist synthesis, the reviewers expect to be able to refine the hypothesized program theory shown in Figure 1.

In addition to synthesizing the evidence for the effectiveness of social accountability interventions, the review will also aim to gain insights into the background of the interventions and the way in which they are defined, designed and evaluated. Guiding questions are:

● What types of social accountability interventions are designed to increase health providers’ and policymakers’ responsiveness? To what extent does the literature distinguish between citizen engagement and citizen oversight strategies?

● What is the underlying perspective or paradigm of the interventions (health systems thinking, rights-based approach, state building and so on)?

● How are social accountability interventions evaluated? Which methods are used and what new questions emerge for content as well as methodology?

### Review process

A realist synthesis, like a standard systematic review, follows a number of steps: defining the scope, objective and research questions; searching for and appraising evidence; extracting and interpreting findings; synthesizing findings and developing conclusions and recommendations [28,36]. This study design is inspired by the published realist synthesis standards and other relevant guidance papers [25,26,28,33].

The synthesis will be conducted in six steps, of which the first has been implemented:

#### Formulating the preliminary program theory

The preliminary program theory presented in this protocol is based on a review of literature and preparatory sessions with the authors and external experts. It is formulated in a broad way with a limited number of predefined assumptions to allow a better understanding of the variety of ways of working of social accountability interventions and responsiveness dynamics. An inductive approach to data collection and analysis will help the reviewers to enrich the preliminary program theory gradually and make the underlying assumptions more explicit. To give some guidance to this inductive process, potential elements of C-M-Os have been defined in Table 1. Later in the synthesis process, the results of previous systematic reviews and literature reviews will, where relevant, be used to contextualize or strengthen the findings and support the review of the hypothesized program theory.

#### Searching and selecting documents

The search for documents will start with a snowball approach whereby references in key review papers mentioned in this protocol will be assessed. This may result in a first selection of relevant documents as well as a refinement of keywords to be used for the database search. A search will be conducted of various academic databases: PubMed, Web of Science, Embase, Scopus and the International Bibliography of the Social Sciences (IBSS). This combination of databases should give a balance between articles focused on (public) health and those focused on broader social and political development. A research librarian will assist in the search. The researchers will look for primary sources (own research), both academic and non-academic (the grey literature, such as evaluation reports, policy documents, dissertations and theses). Papers will include randomized controlled trials, quasi-experimental studies, cohort studies, case studies, surveys and qualitative studies. The terms in Table 2 will be used for the search.

**Table 2:** List of keywords for the search

**Word group 1 (intervention)**	**Word group 2 (setting)**
Citizen, consumer, public, patient, community, user, client, women, men, social, health movement, social movement, rights movement, committee, association, civil society, citizenship	Health, healthcare, health services, health facility, hospital
AND	AND
Engagement, participation, involvement, consultation, representation, advocacy, information, communication, education, sensitization, influence, claiming, agency, mobilization, monitoring, voice, oversight, accountability, negotiation, feedback, complaint, report	Delivery, provision, policymaking, policy formulation, decision-making, program formulation, planning, monitoring, evaluation

A snowball approach will again be used by searching in references in articles and through related citations proposed by the databases. Additional searches will be through Google and professional networks such as the Community of Practitioners on Accountability and Social Action in Health (COPASAH), Equinet, the Affiliated Network of Social Accountability (ANSA) and different Communities of Practice established in the context of the Harmonization for Health in Africa (HHA) initiative. Duplicate references will be filtered out.

A preliminary screening will capture all articles potentially relevant to the review. Relevance refers to the extent to which the reviewers think the document will provide information on elements within the preliminary program theory. This means that titles, abstracts and keywords will be reviewed and, if necessary, full texts, by applying the initial inclusion criteria (see Additional file 1). Documents that meet the criteria will be imported into the Mendeley reference manager. Those that raise doubts will be reviewed by the review team, who will make a final decision. This will further reduce the list of relevant papers. This process will be monitored and documented using a search tree. The selected studies will be organized by type (primary or secondary), relevance, study objective, intervention and setting/location. Studies will then be divided among peer groups consisting of two reviewers. Articles that describe interventions with an explicit aim of strengthening citizen engagement and social accountability in the health sector (inclusion criteria 1a) will be assessed in the first round, followed by those that describe interventions that indirectly relate to citizen engagement and social accountability (inclusion criteria 1b).

#### Extracting the data

The aim of data extraction is to populate the preliminary program theory with evidence [28]. Data extraction will be done using the MaxQDA software, which is used to add codes to texts by highlighting and annotating passages that contain relevant information on the intervention and evidence for the preliminary program theory. A first set of codes will be used for the evidence review questions (intervention, program theory, issues, actors and C-M-Os). A second set of codes will be developed for questions on the evaluation methodology of the studies reviewed (paradigms, viewpoint of evaluators, evaluation methodology, alternative explanations in discussion, new emerging questions and author recommendations). Definitions and examples of concepts are provided in Table 1, they will guide the coding process. For each of these steps, document authors may be contacted if the reviewers wish to obtain additional information.

#### Analyzing and synthesizing the evidence

Per document, a reviewer will document reported evidence and his/her interpretation of the identified C-M-O configurations and related elements (for example, the actors involved and affected). This will be followed by regular peer review sessions of the findings and interpretations per document (see the section on ‘Quality assurance’). Synthesis begins with a theory and ends with a refined theory. This phase will fine-tune the reviewers’ understanding of the interventions; ‘synthesis refers to making progress in explanation’ [36].

In the synthesis phase, the reviewers will try to make sense of the identified C-M-Os by following these recommended steps [26]:

● Identify prominent recurrent patterns of contexts, mechanisms and outcomes (demi-regularities) in the data across the documents and refine C-M-O configurations, with a focus on responsiveness outcomes.

● Confirm or modify the reviewers’ understanding of the demi-regularities based on refined C-M-Os.

● Assess whether and where the C-M-O configurations help to inform the hypothesized program theory. The findings of the review may support or contradict the preliminary theory, underlying assumptions and elements or a combination of both, and will lead to the revision, improvement or refocusing of the program theory or theories.

#### Development of the narrative

The findings will follow the program theory or, more specifically, the underlying hypotheses and review questions. They will include a description of studies and types of intervention, a description of outcomes and a synthesis of the evidence. In the discussion section of the narrative, the findings will be placed in the context of the wider literature on the topic as identified and reviewed in the first stage of the synthesis. The strengths and weaknesses of the methodology and limitations of the study results will also be addressed. There will be a synthesis of the reviewers’ lessons learned with regard to the use of realist synthesis for evidence building in this field of work.

#### Dissemination and knowledge-sharing strategy

Throughout the process, practitioners and researchers will be involved from the research institutes that participate in this review. In addition, colleagues from the larger research program on social accountability in maternal health service delivery will be involved, in particular in Burundi, the Democratic Republic of Congo, Mali and Guinea. Opportunities will be sought to share and discuss the results and in particular those that report on post-conflict or fragile settings. On these occasions, the integration of results and lessons in the programs of the respective organizations will be discussed. This study belongs to a broader research field of social accountability, participation and citizen-centered development. To encourage the use of the insights gained through this synthesis, the reviewers will: (1) integrate the lessons and the refined theory in their own research program, (2) share results with international networks of researchers and practitioners such as the ones mentioned above and (3) present the approach and results at conferences and seminars in the Netherlands and elsewhere.

### Review team

The following team will conduct the review: EL (lead investigator, search and initial selection of literature, coordination of data extraction, joint analysis and synthesis, narrative development and drafting the report), MD and BG (data extraction, participation in joint analysis and synthesis, narrative development and supporting the drafting of the report), and JB (supervision of the research and providing feedback on the synthesis and the report).

### Quality assurance

Quality is dependent upon the reviewers’ explicitness and capacity for reflection [28]. Three measures will be adopted to ensure quality. First, all retrieved articles will be assessed by two reviewers separately. After every fifth article, the reviewers will revisit their findings together to reach agreement. If there is no consensus, the questions will be discussed with the review team and any disagreement will be resolved. All joint decisions and changes in the process will be documented and, later, synthesized. Second, reviewers will use an online logbook to document their individual and joint actions, dilemmas and decisions. At regular intervals, the team will meet to discuss these logbooks, as well as the successes and difficulties encountered. Finally, throughout the review, the team will hold small group discussions and reflection sessions, which will build in checks and balances and enhance team learning, as some reviewers are more experienced with realist synthesis than others.

## Discussion

### Crossing disciplines and paradigms

An often cited difficulty with evaluating participatory programs is when concepts are not well defined or defined differently, which makes comparison complicated. Social accountability is an umbrella term and the application of concepts and methods varies enormously, as well as the goals set, the actors who initiate and participate in the processes, the levels of action and timing, and the topical focus. The various uses of the term is largely related to the underlying paradigms of the interventions. For example, the previously mentioned institutionalist and social movement perspectives are often presented as being opposed to each other, which may lead to an underestimation of the broad range of interventions and the knowledge they generate.

In this review, the team would like to address these challenges. Firstly, the team will try to overcome the paradigmatic dichotomy by using a framework that builds on both institutionalist (that is, system thinking and institutional design) and social movement (that is, empowerment theory) perspectives by obtaining sources from different databases. Secondly, instead of testing a definition, in this review the questions are treated as empirical ones asking whether and how particular approaches lead to certain kinds of change in particular contexts, regardless of the intervention paradigm. The team members, however, also recognize the inherent difficulty of this open approach. During the analysis, the team will probably be confronted with types of blended interventions that intentionally or unintentionally have both institutional and empowering outcomes. To make sense of this puzzle, and as can be read in the methods section, the team will use two rounds of selection and analysis: the first will cover the interventions that the reviewers expect to inform the program theory directly, and the second will cover the more implicit or blended interventions that can be used to further contextualize the direct approaches.

### Complementing previous reviews

Recent studies have mapped interventions and outcomes for participation and accountability [6,14,37,38]. Gaventa and Barrett (2010) mapped the outcomes, such as citizenship and responsiveness, of 100 case studies. Although they gained insights into important contextual factors, they concluded that a more in-depth analysis was needed to explain better what makes an intervention successful [9]. Similarly, Marston reviewed evidence from four well-known community participation initiatives in Bangladesh, two of which were considered successful and two of which were not. Despite some guesses regarding the role of contextual factors, the authors emphasized that the underlying reasons for success or failure of the interventions were yet to be uncovered. To the best of our knowledge, this is the first attempt to use a realist approach to synthesize literature on governance, accountability and citizen participation. The reviewers expect this review will not only offer new insights on social accountability interventions but also on the added value of this approach to synthesis within the social science and public health research community.

### Internal and external validity

To strengthen the internal validity, it will be important during data extraction to check on a regular basis the abstracted data against the data and arguments in original article to ensure that the analysis does not deviate from the original intent of the authors [39]. Realist synthesis requires continuous assessment and a comparison of findings from different perspectives. The collective scrutiny of inquiry and regular team discussions will enhance the validity and coherence of the inferences made [29]. Realist synthesis is theory based, which increases its external validity and the potential of this review to inform practice and policy. The MRT that will evolve from this study may be used across contexts.

## Abbreviations

C-M-O: Context-mechanism-outcome; CSO: Civil society organization; MDG: Millennium Development Goal; MRT: Middle-range theory; NGO: Non-governmental organization.

## Competing interests

The authors declare that they have no competing interests.

## Authors’ contributions

All authors agreed on the need for a study protocol. EL conducted the literature review, drafted the preliminary program theory, designed the review and coordinated the feedback for different versions of the manuscript. MD participated in the drafting of the preliminary program theory and the review design and helped to draft the manuscript. BG and JB gave advice regarding the review design and helped to draft the manuscript. All authors read and approved the revised and final versions of the manuscript.

## Supplementary Material

Additional file 1 Inclusion and exclusion criteria. Click here for file
