# Supplementary material for: A realist synthesis of the effect of social accountability interventions on health service providers’ and policymakers’ responsiveness
Source: Syst Rev. 2013 Nov 7;2:98. doi: 10.1186/2046-4053-2-98 (PMC4226265; doi:10.1186/2046-4053-2-98)
Supplement: Additional file 1 — Inclusion and exclusion criteria. [file 2046-4053-2-98-S1.docx]

**Additional file 1. Inclusion and exclusion criteria**

**Inclusion criteria**

1. *Relevance*
2. The document addresses interventions or reforms that aim *explicitly* (in their objectives) to strengthen citizen engagement or social accountability in the health sector (health service delivery and/or health policymaking processes) or the document describes opinions on, and experiences with, citizen engagement or social accountability in the health sector (case study, descriptive study)
3. The document addresses interventions that more *implicitly* (in the process or outcomes) relates to citizen engagement and social accountability in the health sector. In these cases, the studies will only be included if they report on voice and/or responsiveness or relational issues.

*For further descriptions of types and relevance of interventions, see appendix 1.*

1. *Setting*
2. The document deals with at least one country with medium to low levels of governance capacity and quality. The list of countries is derived from the Worldwide Governance Indicators (WGI) database from the World Bank (http://info.worldbank.org/governance/wgi/sc_country.asp)
3. The document deals with interventions at local (community, district) or national level
4. *Type and characteristics of studies*
5. The document is based on primary research (additional code “P”)
6. Documents will have been developed/published between 2003 and 2013
7. English, French and Spanish language
8. For grey literature the credibility and quality will be assessed, criteria:
   - Recognizable research process, not just anecdotal reporting
   - Independence of the author (if from the organization, a level of external peer review should have taken place)
   - Contact details of organization or authors are provided

**Exclusion criteria**

1. The document is not about the health sector
2. The intervention has a global character (e.g. global mobilization campaigns etc.)
3. The document is based on secondary research (additional code “S”) or is a conceptual paper

The documents that respond to criteria 1b and criteria k are not yet included in the first round of review but may be included at the end of the review, to enrich findings, in particular in the discussion section.
